# Phosphatidylethanol versus clinical criteria to diagnose alcohol use relapse after liver transplantation: a prospective cohort study

**DOI:** 10.1007/s12072-026-11094-4

**Published:** 2026-05-22

**Authors:** Lubomir Skladany, Svetlana Adamcova Selcanova, Daniela Zilincanova, Jana Čiefová, Daniel Jan Havaj, Karolina Kristina Sulejova, Natalia Kubanek, Peter Secnik, Jana Zemlickova, Tomas Koller, Juan Pablo Arab

**Affiliations:** 1 HEGITO - Div Hepatology, Gastroenterology and Liver Transplant, Dept. Internal Medicine 2, Faculty of Medicine, Slovak Medical University, F. D. Roosevelt University Hospital, Banska Bystrica, Slovakia; 2 2nd Department of Internal Medicine, L. Pasteur University Hospital and PJ Safarik University, Kosice, Slovakia; 3 SK-LAB, s.r.o. - Clinical Laboratory, Lucenec, Slovakia; 4 Sanatorium AT, Specialized Psychotherapeutic Treatment Facility, Bratislava, Slovakia; 5 Gastroenterology and Hepatology Subdivision, 5th Department of Internal Medicine, Comenius University Faculty of Medicine, University Hospital Bratislava, Bratislava, Slovakia; 6 Division of Gastroenterology, Hepatology, and Nutrition, Department of Internal Medicine, Stravitz-Sanyal Institute of Liver Disease and Metabolic Health, Virginia Commonwealth University School of Medicine, Richmond, USA; 7 Departamento de Gastroenterologia, Escuela de Medicina, Pontificia Universidad Catolica de Chile, Santiago, Chile

**Keywords:** Alcohol-associated liver disease, Liver transplantation, Phosphatidylethanol, Relapse

## Abstract

**Background:**

Alcohol-associated liver disease (ALD) with concomitant alcohol use disorder (AUD) is a leading indication for liver transplantation (LT). Return to alcohol use after LT (RAULT) affects a considerable proportion of recipients but lacks an objective diagnostic standard. We evaluated blood phosphatidylethanol (PEth) as a direct biomarker of RAULT and compared it with conventional clinical criteria (ClinC).

**Methods:**

We prospectively studied adult patients who underwent LT for ALD between 2008 and 2023. Since 2020, RAULT has been assessed quarterly using both ClinC and PEth (cut-off > 0.05 µg/L). Discordant results were interpreted using PEth as a biochemical comparator, given its higher objectivity and specificity compared with ClinC, although it was not considered a definitive diagnostic standard.

**Results:**

Among 143 patients (median age 56 years; 29% female; median post-LT follow-up 72 months), during a 3-year comparative study interval, RAULT was identified in 46 patients (32.2%) by PEth and in 69 patients (48.3%) by ClinC. *Concordance* between the two methods was observed in 98 cases (68.5%): in 63 cases (44.0%) for absence of RAULT and in 35 cases (24.5%) for presence of RAULT. *Discordance* occurred in 45 cases (31.5%), including 34 ClinC-positive/PEth-negative cases (23.8%) and 11 ClinC-negative/PEth-positive cases (7.7%). Quarterly and semi-annual PEth testing yielded comparable overall RAULT detection rates; however, quarterly testing identified a higher proportion of severe RAULT (16.1% vs. 12.6%). Time since LT independently predicted RAULT according to PEth (OR = 1.17; 95% CI 1.08–1.27; p = 0.0002).

**Conclusions:**

Clinical criteria may overestimate the occurrence of RAULT after liver transplantation. Quarterly or semi-annual PEth testing provides additional objective insight into post-transplant alcohol use dynamics and may enhance monitoring of patients with AUD following LT.

**Supplementary Information:**

The online version contains supplementary material available at 10.1007/s12072-026-11094-4.

## Introduction

In Central Europe, alcohol-associated liver disease (ALD) remains the leading cause of liver-related mortality and continues to represent a major indication for liver transplantation (LT) [1, 2]. Eligibility criteria remain heterogeneous across countries and transplant centers. In some regions, a fixed period of alcohol abstinence—most commonly six months—is strictly required prior to listing, commonly referred to as the “6-month rule.” In contrast, other programs apply more individualized psychosocial assessments without a predefined abstinence duration, relying instead on multidisciplinary evaluations of relapse risk [3–5]. Sustained abstinence is the most critical determinant of long-term prognosis in ALD, both before and after LT [6–8]. Recent evidence further supports its central role in determining outcomes. In addition, early identification of relapse, appropriate therapeutic intervention, and return to abstinence may result in comparably favorable long-term outcomes [9].

While ALD represents a leading indication for LT in several regions, the sociocultural context substantially influences both its prevalence and post-transplant outcomes. In Central and Eastern European countries, including Slovakia, structural determinants—such as economic hardship, high per capita alcohol consumption, limited access to specialized addiction services, and persistent stigma surrounding mental health—may contribute to a disproportionately high ALD burden and increased relapse risk. In contrast, in certain Asian countries where alcohol consumption is less socially normalized, ALD accounts for a comparatively smaller proportion of liver transplant indications. These differences likely reflect broader variations in healthcare systems, public health infrastructure, and societal attitudes toward addiction [10, 11].

Reported RAULT rates vary widely, ranging from 7 to 95%, depending on multiple factors, including diagnostic criteria and duration of follow-up [12, 14]. Our group previously reported a 26% incidence of RAULT based on clinical criteria (ClinC) over nearly 10 years of follow-up [1].

ClinC, as described by Pageaux et al., integrates the clinician’s impression of resumed alcohol use, reports from the patient or close relatives, and at least two indirect laboratory markers: an AST/ALT ratio > 1, gamma-glutamyltransferase (GGT) exceeding the upper limit of normal (ULN), and mean corpuscular volume (MCV) above ULN [1].

However, in the absence of an objective reference standard, diagnosing RAULT remains challenging. Contributing factors include patient denial driven by stigma, the subjective nature of clinical impressions—particularly in individuals with a history of relapsing alcohol use—and the limited sensitivity and specificity of indirect biochemical markers [15, 17]. Misattributing abnormal laboratory values to RAULT may not only reinforce stigma but also delay recognition of other post-LT complications, such as graft rejection, drug toxicity, or infection [18–20].

A validated, reliable, and accessible direct biomarker with sufficient backward alcohol capture time could significantly improve post-LT surveillance and potentially enhance clinical outcomes. One of the most promising candidates is phosphatidylethanol (PEth), first described by Alling et al. in 1983 [21–26]

PEth is formed within red blood cell membranes and other tissues through a transphosphatidylation reaction catalyzed by phospholipase D following ethanol ingestion [24, 27, 28]. With an elimination half-life of approximately four days, PEth remains detectable in blood for up to 2–4 weeks after alcohol intake, making it one of the direct alcohol biomarkers with the longest detection windows currently used in clinical practice.

PEth concentrations may be influenced by several factors, including the amount and duration of alcohol consumption, red blood cell turnover (e.g., recent transfusions), liver function, body composition, and metabolic rate [29, 30]. Its reported sensitivity (97–100%) and specificity (66–96%) indicate that PEth is a highly reliable biomarker for both episodic and chronic alcohol use. It may also serve as an indicator of abstinence when using a cutoff value of < 0.05 µg/L [31, 32].

Notwithstanding its diagnostic utility, the broader implementation of PEth testing depends on regional availability, laboratory infrastructure, and associated costs.

The primary objective of this study was to compare the detection rate of RAULT using two diagnostic approaches: PEth and ClinC.

Given the absence of a universally accepted reference standard for RAULT, this comparison was designed to assess the level of concordance and discordance between PEth and ClinC, while acknowledging the methodological limitations inherent to both approaches.

Secondary objectives were to compare the diagnostic yield of quarterly versus semi-annual testing, to assess the severity of RAULT based on predefined PEth concentration thresholds, and to identify independent predictors of RAULT [1, 31–33].

## Patients and methods

This prospective, single-center study enrolled consecutive adult patients who underwent LT for ALD between May 2008 and June 2023. All patients received deceased donor liver transplantation (DDLT), as living donor liver transplantation (LDLT) was not available during the study period. Patients who died within the first month post-LT or were younger than 18 years at the time of transplantation were excluded.

Baseline demographic and clinical data were collected, including age, sex, pre-transplant Model for End-Stage Liver Disease Sodium (MELD-Na) score, and Child–Pugh score (CTP). Since July 2020, patients were evaluated quarterly for subjective and objective indicators of alcohol consumption, potential relapse risk factors (see below), and laboratory parameters, including mean corpuscular volume (MCV), aminotransferase levels, and PEth concentrations.

Although the study cohort included patients transplanted between 2008 and 2023, both ClinC and PEth assessments were applied in parallel starting in June 2020. Thus, all comparative analyses between ClinC and PEth were restricted to this shared timeframe, thereby minimizing the risk of lead in bias.

Moreover, we systematically reviewed all cases classified as RAULT to assess for alternative or concurrent diagnoses. None of the RAULT cases were attributable to graft rejection, drug-induced liver injury (DILI), or active infection at the time of diagnosis. Comprehensive laboratory, microbiological, and imaging evaluations were performed when clinically indicated, and cases with diagnostic uncertainty were reviewed by a multidisciplinary transplant team.

While ClinC criteria may lack specificity, our structured diagnostic approach was designed to minimize misclassification related to overlapping post-LT complications.

## RAULT by ClinC

The clinical diagnosis of RAULT using ClinC was based on the method described by Pageaux et al. ClinC has been used as the standard relapse assessment method at our center since 2008 for all patients. Other validated instruments, such as the Timeline Follow-Back (TLFB), were not part of routine clinical practice and could not be applied retrospectively [34].

The “clinician’s impression” reflects the hepatologist’s integrated judgment based on patient interviews, collateral objective information, and the overall clinical context. We acknowledge that ClinC is an indirect and nonspecific approach and has not been formally validated against standardized alcohol-use assessment tools.

RAULT was also diagnosed in the presence of any positive blood alcohol test or the fulfillment of at least one of the following criteria for RAULT and at least two criteria for severe RAULT:

- Clinician’s impression that the patient had resumed alcohol use and/or patient-reported alcohol consumption
- Alcohol use reported by a cohabiting family member or close proxy
- At least two laboratory abnormalities among: AST/ALT ratio > 1 with AST > ULN; GGT > ULN; MCV > ULN [30] at any sampling time-point

## RAULT by PEth

A PEth cut-off ≥ 0.05 µg/L was considered sufficient for diagnosing RAULT, and ≥ 0.3 µg/L for severe RAULT, regardless of patient- or clinician-reported alcohol use according to ClinC.

PEth samples were collected during routine outpatient visits, concurrently with clinical assessments. RAULT rates were recorded using both ClinC and PEth, and concordance and discordance between the two methods were evaluated.

Discordant findings were categorized as “unveiled” RAULT (ClinC negative, PEth positive) or “vindicated” RAULT (ClinC positive, PEth negative), based on PEth results given its objectivity and high specificity. However, in the absence of a universally accepted reference standard, the interpretive value of PEth was assessed within the broader clinical context. (Table 1). To assess relapse severity, we applied the classification systems proposed by Lucey (for ClinC-based RAULT) and by the World Health Organization (for PEth-based RAULT), (Table 2, Supplementary Table S1) 35–37].

**Table 1:** Classification of diagnostic concordance and discordance between PEth and ClinC

Label	RAULT by ClinC	RAULT by PEth
RAULT, concordance	Positive	Positive
Sobriety, concordance	Negative	Negative
RAULT, discordance *(RAULT not diagnosed by ClinC but unveiled by PEth)*	Negative	Positive
Sobriety, discordance *(PEth vindicated patients of RAULT diagnosed by ClinC)*	Positive	Negative

*RAULT* Relapse of alcohol use after liver transplantation, *ClinC* Clinical criteria, *PEth* Phosphatidylethanol, Concordance: Agreement between RAULTs by ClinC and PEth, Discordance ClinC and PEth were at odds

**Table 2:** Severity thresholds for RAULT by ClinC and PEth criteria

	Definition		
ClinC	Occasional drinking (slip-up)		Consumption of a limited amount of alcohol, followed by re-established abstinence
	Harmful (addictive) drinking		Consumption of four or more drinks in a day or for 4 or more days in succession
PEth (ug/l)	Alcohol consumption (average/day)		Categories of alcohol consumption (NIAAA – National Institute of Alcohol Abuse and Alcoholism)
	< 0.05	Men: < 40 g Women: < 30 g	Low or no consumption
	0.05 -0.3	Men: 40—60 g Women: 30—60 g	Moderate consumption
	> 0.3 µg/l	Men: > 60 g Women: > 60 g	Heavy/severe consumption

*NIAAA* National institute of alcohol abuse and alcoholism*, PEth* Phosphatidylethanol, *RAULT* Relapse of alcohol use after liver transplantation, *ClinC* Clinical criteria

## PEth analysis and laboratory standards

PEth was measured using high-performance liquid chromatography coupled with tandem mass spectrometry (HPLC–MS/MS). The in-house analytical protocol was adapted from the method described by Helander et al. [27]. All analyses were performed in an ISO 15189-accredited laboratory in accordance with standardized validation and quality control procedures to ensure linearity, precision, control of systematic bias, and overall analytical accuracy. Validation procedures followed Clinical and Laboratory Standards Institute (CLSI) guidelines EP05-A3 and EP15-A3 [38].

During the study period, all patients were informed in advance about PEth testing and provided written informed consent. This approach minimized the risk of unrecorded relapses due to sample refusal, which is sometimes regarded in addiction medicine as an indirect indicator of ongoing alcohol use.

To inform future cost-effectiveness analyses, we compared the diagnostic yield of RAULT detection by PEth using quarterly versus semi-annual testing intervals.

## Risk factors and statistical analysis

Potential risk factors for RAULT were prospectively recorded, including time since LT, age, smoking status, history of treated psychopathology, noncompliance with follow-up visits, absence or loss of a life partner, adverse social circumstances (e.g., divorce, financial hardship), and unemployment [39–41]. However, the scope of our study was limited to severe psychiatric disorders that could constitute a contraindication to listing the patient for liver transplantation. Statistical analyses were conducted using MedCalc version 23 (MedCalc Software Ltd., Belgium) and R statistical software (www.r-project.org). Continuous variables were reported as medians with interquartile ranges (25th–75th percentiles), and categorical variables as counts and percentages. Group comparisons were performed using the nonparametric Mann–Whitney U test for continuous variables and the chi-square test for categorical variables. To identify independent predictors of any RAULT and of severe RAULT, we constructed multivariate logistic regression models adjusting for age, sex, follow-up compliance, smoking, pre-transplant hepatic encephalopathy, partner status, employment status, history of psychopathology, and duration of post-LT hospitalization. Statistical significance was defined as a two-tailed p-value < 0.05.

**Ethical considerations**

All procedures involving human participants were conducted in accordance with the ethical standards of the institutional research committee of F. D. Roosevelt Teaching Hospital (approval dated July 15, 2019), the 1964 *Declaration of Helsinki* and its later amendments, and the *Declaration of Istanbul* on Organ Trafficking and Transplant Tourism. Written informed consent was obtained from all participants prior to transplantation, and participants agreed to the use of their data for research purposes. Participants’ anonymity and confidentiality were strictly maintained.

## Results

Of the 360 patients who underwent LT during the 72-month study period, 143 (40%) received LT for ALD. Among the remaining patients, 175 (49%) were transplanted for non-ALD etiologies, and 42 were excluded due to a follow-up period of less than one month.

The study cohort had a median age of 56 years (IQR: 48–61), and 29% were female. The median MELD-Na and CTP scores at the time of LT were 15.6 (IQR: 13.0–18.7) and 9.0 (IQR: 8.0–11.0), respectively. Summary statistics and baseline characteristics are presented in Table 3.

**Table 3:** Baseline demographic and clinical characteristics of the study cohort

Parameter	N = 143
	n (%) or median [25, 75 percentile]
Age, years	55.81 [48.41, 61.17]
Sex, n (%)	
male	101 (70.6)
female	42 (29.4)
Pre-LT status	
Liver disease etiology	
Alcohol	117 (81.8)
Alcohol and HBV	1 ( 0.7)
Alcohol and HCV	2 ( 1.4)
Met-ALD	23 (16.1)
Body mass index, kg/m^2^	26.90 [23.98, 30.05]
Child–Pugh score	9.00 [8.00, 11.00]
Child–Pugh stage	
A	5 ( 3.5)
B	69 (48.3)
C	69 (48.3)
MELD-NA score	15.57 [13.00, 18.73]
Serum albumin, (35—52 g/l)	30.00 [27.00, 34.00]
International normalized ratio	1.49 [1.32, 1.67]
Serum bilirubin, (0—21 umo/l)	44.00 [30.75, 69.20]
Serum creatitnine,(49—90 umol/l)	78.00 [63.00, 100.00]
Days on the waiting list, d	36.00 [12.00, 131.50]
Post LT status	
Days in hospital post-LT	22.00 [17.00, 32.00]
Acute graft rejection, n (%)	5 ( 3.5)
Social status, n (%)	
retired	55 (38.5)
invalidity	47 (32.9)
unemployed	13 ( 9.1)
employed	27 (18.9)
Compliance with post-LT controls, n (%)	138 (96.5)
Diagnosed psychopathology, n (%)	1 ( 0.7)
No life—partner, n (%)	38 (26.6)
Smoking, n (%)	34 (23.8)
Weeks from LT (follow-up)	327.57 [138.50, 526.86]
Months from 1st to last PEth and ClinC evaluation	22,3 [21.6, 24.7]
Number of PEth test days	5.00 [4.00, 6.00]
RAULT	
RAULT by ClinC, n (%)	69 (48.3)
Severe RAULT by ClinC, n (%)	20 (14.0); *28.9% of RAULT by ClinC*
RAULT by PEth, n (%)	46 (32.2)
Severe RAULT by PEth, n (%)	23 (16.1) *50% of RAULT by PEth*
Time-to-positive PEth (days, any relapse)	140 [58, 359]

*ALD* alcohol-associated liver disease, *ClinC* clinical diagnostic criteria of RAULT, *HBV* hepatitis B virus infection, *HCV* Hepatitis C virus infection, LT *liver transplantation*, *MetALD* New definition, combination of ALD with metabolic dysfunction-associated steatotic liver disease (MASLD), *MELD Na* Model for End Stage Liver Disease – Natrium, *PEth* phosphatidyl ethanol, RAULT relapse of alcohol use after liver transplantation

### RAULT by ClinC

**Fig. 1:**
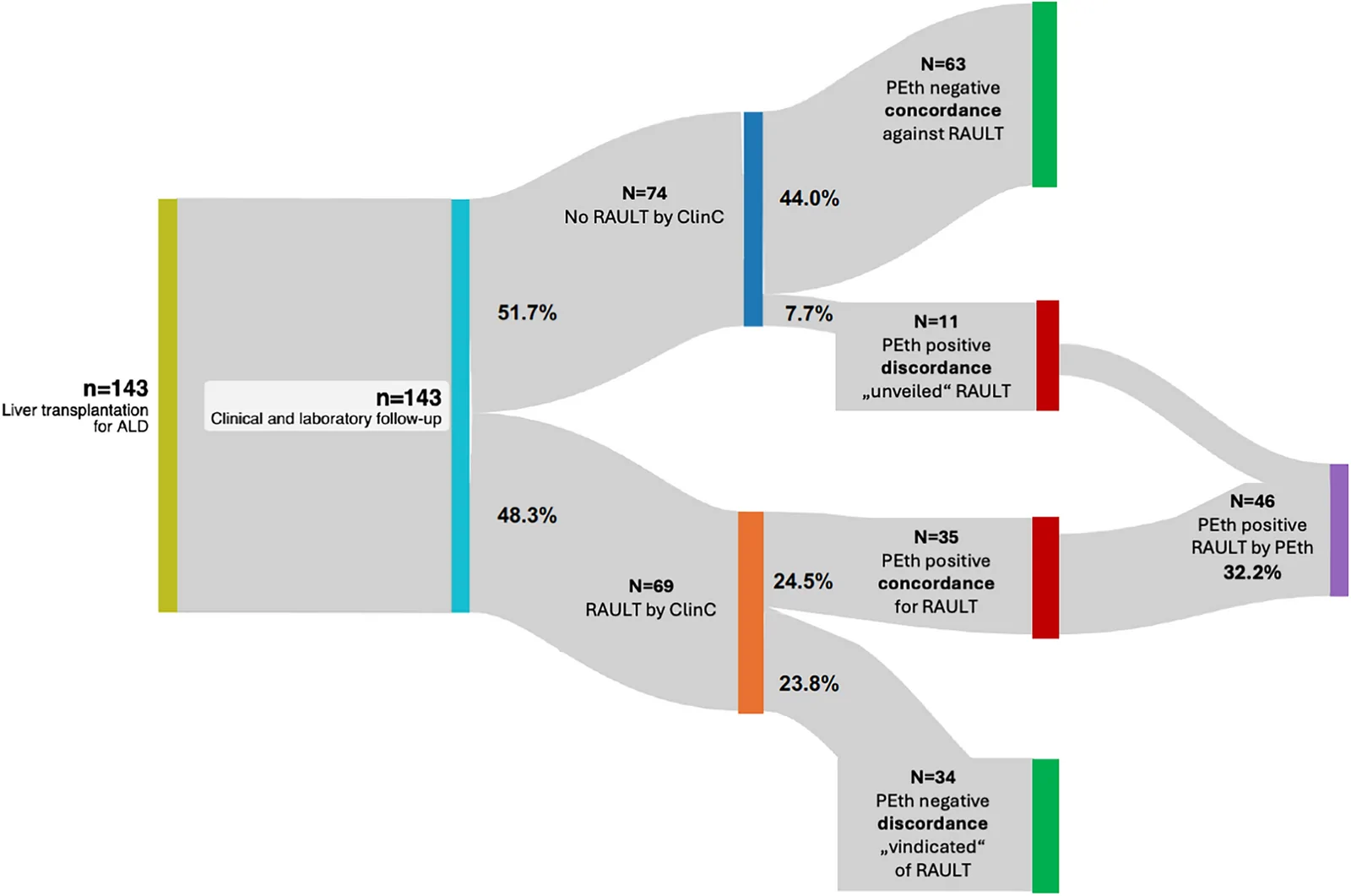
Use of phosphatidylethanol versus clinical criteria to diagnose alcohol relapse after liver transplantation: A prospective cohort study

RAULT by ClinC was diagnosed in 69 of 143 patients (48.3%). Of these, 35 patients (24.5%) had concordant positive PEth results, confirming RAULT by PEth. The remaining 34 (23.8%) had negative PEth results, indicating discordant findings between ClinC and PEth in 49.3% of ClincC positive cases. Among the 74 patients (51.7%) in whom RAULT was not suspected by ClinC, 63 (44.0%) had concordant negative PEth results, with both methods yielding negative findings. In contrast, 11 patients (7.7%) had positive PEth results when RAULT was not suspected by ClinC (Fig. 1). In total, ClinC and PEth results were concordant in 98 of 143 patients (68.5%) and discordant in 45 (31.5%). (Fig. 1, Table 4).

**Table 4:** Diagnostic concordance and discordance between ClinC and PEth in detecting RAULT

	RAULT
RAULT, concordance	50.7%
Sobriety, concordance	85%
RAULT, discordance *RAULT was not diagnosed by ClinC but was unveiled by PEth*	15%
Sobriety, discordance *PEth vindicated patients from RAULT diagnosed by ClinC*	49.3%

*RAULT* Relapse of alcohol use after liver transplantatio, *ClinC* Clinical criteria*, PEth* Phosphatidylethanol, *Concordance* Diagnosis by ClinC and PEth agreed*, Discordance ClinC and PEth were at odds, PEth was considered the gold standard*

### RAULT by PEth

We found evidence of RAULT by PEth in 46 of 143 patients (32.2%). Based on quarterly PEth monitoring, we found evidence of a single episode in 8 patients (5.6%), two episodes in 8 (5.6%), three episodes in 4 (2.8%), four episodes in 8 (5.6%), five episodes in 9 (6.3%), six episodes in 6 (4.2%), seven episodes in 2 (1.4%), and eight episodes in 1 patient (0.7%) (Supplementary Table S2).

### Severe RAULT

Severe RAULT by ClinC was diagnosed in 20 of 143 patients (14% of the total cohort and 28.9% of those with RAULT). Among those, 16 patients also tested positive for PEth, corresponding to 80% concordance between ClinC and PEth. In comparison, 4 patients had negative PEth (20% discordance). Among the 123 patients (86%) not diagnosed with severe RAULT by ClinC, 116 (94.3%) had concordant negative PEth, confirming abstinence, whereas 7 patients (5.7%) had positive PEth, indicating severe RAULT undetected by ClinC. Using PEth as a biochemical benchmark, ClinC would overestimate severe RAULT by 20% (4/20) with a false-negative rate of 5.7% (7/123) (Supplementary Table S3; Supplementary Table S4). Severe RAULT, as defined by PEth criteria, was observed in 23 patients (16.1% of the cohort), representing 50% of all RAULT cases diagnosed by PEth.

### Sex-based comparisons of RAULT diagnosed by PEth and by ClinC

Sex-based comparisons did not demonstrate statistically significant differences in PEth-defined relapse. The proportion of relapse according to PEth was 30.69% in men and 35.71% in women (p = 0.561). Similarly, the rate of severe PEth-defined relapse was comparable between men and women (14.85% vs. 19.05%, respectively; p = 0.537). In contrast, the relapse defined by the ClinC was numerically more frequent in men than women (53.47% vs. 35.71%), although the difference did not reach statistical significance (p = 0.054).

### Sampling frequency and RAULT capture-rate

The median PEth concentrations across consecutive quarterly samplings, stratified by RAULT category are presented in Supplementary Figure S1. When comparing RAULT prevalence by sampling frequency (Table 5), RAULT by PEth was diagnosed in 46 patients (32.2%) with quarterly testing and in 43 patients (30.1%) with semi-annual testing (*p* = 0.075). However, quarterly sampling of PEth identified more cases of severe RAULT than semi-annual sampling (16.1% vs. 12.6%, *p* = 0.018). Supplementary Table S5 summarizes the clinical and demographic characteristics of patients with and without RAULT by PEth.

**Table 5:** Comparison of RAULT detection rates by PEth between quarterly vs. semi-annual sampling

	Quarterly PEth sampling	Semi-annual sampling	P-value
N = 143	N (%)		
Any RAULT, PEth > 0.05	46 (32.2%)	43 (30.1%)	P = 0.075
Severe RAULT, PEth > 0.3	23 (16.1%)	18 (12.6%)	P = 0.018

### Risk factors for RAULT

In multivariate logistic regression a single independent predictor of RAULT by PEth was time (in years) since LT (OR = 1.171, 95%CI 1.077–1.273, *p* = 0.0002), and likewise for severe RAULT (OR = 1.142, 95% CI 1.033–1.262, *p* = 0.0093).

## Discussion

This prospective study compared the rate of detection of return to drinking after liver transplantation (RAULT) by ClinC and PEth. Using Clin-C and PEth, RAULT was identified in 48.3% and 32.2% of patients respectively. We interpret our findings as a comparison between two distinct diagnostic approaches. In more than two-thirds of cases, both modalities were concordant, whereas approximately one-third demonstrated discordant results. Among patients classified as having RAULT by ClinC, 49.3% were not corroborated by PEth. Conversely, PEth was positive in approximately 15% of cases classified as negative by ClinC.

The PEth-defined relapse rate was not influenced by sex. Semi-annual PEth testing identified a comparable overall rate of RAULT but was less sensitive in detecting severe RAULT. Time since LT emerged as the only independent predictor of both overall and severe RAULT.

Overall, ClinC identified more cases of RAULT than PEth, suggesting possible overestimation by clinical criteria. However, these findings should be interpreted cautiously given the absence of a universally accepted standard for RAULT and the inherent limitations of both diagnostic approaches.

### RAULT and concordance/discordance of diagnostic tools

In our study, the prevalence of RAULT was high but comparable to previously published reports [1, 2, 6, 8]. It is well established that the sensitivity of PEth is limited to a 2–4-week detection window and may be influenced by red blood cell turnover, blood transfusions, or interindividual metabolic variability. Originally, PEth has been proposed to resolve diagnostic uncertainties associated with traditional ClinC-based assessments. PEth has been validated as a sensitive marker of recent alcohol use, including in the transplant setting [25, 32]. In the pre-transplant and post-transplant monitoring, the use of PEth has been demonstrated by Barrio et al. and De La Torre et al. [42, 43]. Our findings are consistent with prior work by Fleming et al., who demonstrated the value of serial PEth monitoring in post-LT populations [31]. Winder et al. [44] also reported discordance between ClinC and PEth assigned RAULT cases. Fipps et al. reported a high rate of mismatch among patients evaluated for liver transplant [45], while Segal et al. emphasized the limited concordance between self-reported alcohol use and PEth [46]. Similarly, Scholten et al. found substantial disagreement between PEth levels and patient declarations in chronic liver disease populations [47].

In contrast, the diagnostic performance of ClinC varies and can be confounded by comorbidities, behavioral history, and confirmation bias, where clinicians may over-attribute nonspecific findings to alcohol use [15, 16, 33]. In most ClinC-positive / PEth-negative cases, discordance might have been driven by nonspecific laboratory abnormalities or clinical suspicion rather than patient- or caregiver-reported alcohol use. Some of the abnormalities in GGT, AST to ALT ratio, or MCV in the post liver-transplant setting may also be unrelated to alcohol use. Thus, we believe that the discordances may reflect over-diagnosis by ClinC rather than a definitive misdiagnosis. More sensitive approaches to systemize detection of alcohol use in the LT context have been proposed, such as TLFB [9]. However, even ClinC has not been validated against TLFB.

We must underscore that the diagnosis of RAULT remains challenging. Without PEth, we should be reminded of “*in dubio pro reo”*, despite the well-known tendency of some patients with ALD and AUD to underreport or conceal alcohol use [16, 48]. The use of PEth can strengthen the “*rapport”* and support additional dialogue with the patient, family members, and addictologists aiming to achieve concordance of the available evidence with the post-LT reality. This approach may minimize the stigma, a very serious issue harming patients with ALD/AUD (18, 48–51). Moreover, negative PEth may also lead us to consider other alternatives of abnormal clinical or laboratory findings having a possible impact on the prognosis [49].

Lastly, our findings underscore the need for comprehensive post-LT addiction management, including both behavioral and pharmacological interventions, which remain limited in many healthcare systems. Although pharmacological agents such as disulfiram, nalmefene, and acamprosate have demonstrated efficacy in relapse prevention following alcohol detoxification, these medications were not systematically used in our cohort.

At the time of the study, integrated addiction care was not systematically established in Slovakia, which may have limited access to post-LT pharmacotherapy for alcohol use disorder (MAUD). However, efforts to develop multidisciplinary addiction services are currently underway, and addiction specialists have recently been incorporated into long-term follow-up protocols.

### Sampling frequency and RAULT detection

Our comparison of quarterly vs. semi-annual testing showed comparable detection rates of RAULT while quarterly testing has demonstrated a significantly better capture of severe RAULT. This suggests that semi-annual testing may be appropriate for low-risk patients, while quarterly monitoring should be prioritized in high-risk scenarios. Risk stratification can be based on the complex addictological examinations or on PEth dynamics over time. Low-risk patients would be defined as those who either (i) had consistently negative PEth results, or (ii) had a single isolated positive PEth result with a concentration < 0.3 µmol/L, consistent with mild or incidental alcohol exposure. High-risk patients would be defined by repeated positive PEth tests (≥ 2 episodes) or PEth levels exceeding 0.3 µmol/L, indicating sustained or heavy alcohol use. As shown in Supplementary Fig. 2 and Table S1, most patients in the low-risk category had either no positive PEth during follow-up or only one mild elevation. Conversely, patients in the high-risk group showed recurrent or escalating PEth levels, often exceeding the threshold for moderate-to-severe relapse. These findings support a tailored approach to monitoring frequency. Interestingly, median PEth concentrations remained stable over time, indicating consistent behavioral patterns, whether abstinent or relapsing, among many patients.

### Risk factors for RAULT

The association between the time after LT and the rate of relapse should be interpreted cautiously due to the relatively short median follow-up. However, it is consistent with the findings of Yu et al. who observed increasing relapse rates over time (12% at one year; 19% at three years) [52]. This pattern aligns with previous reports suggesting that the protective effect of pre-transplant abstinence may diminish with time [12, 53]. Other studies have identified predictors such as smoking, social isolation, and low socioeconomic status [41, 54, 55]. Our previous report also highlighted smoking and bereavement as relevant factors [1]. Egawa et al. identified follow-up noncompliance and smoking as risk factors [55], while Kelly et al. only a pre-transplant tobacco use [54]. Younger age has been variably associated with increased risk, though not consistently [56–60]. In contrast, social support has emerged as a consistent protective factor [59, 61, 62]. It should be emphasized, that the studies are confounded by heterogeneity of RAULT definitions, diagnostic methods, duration of follow-up and socio-cultural contexts [13, 33].

### Limitations

Our study has several limitations. First, it was conducted at a single center with a high proportion of ALD indications for LT, which may limit generalizability. However, as the single transplant center in the country, our liver-transplant registry provided robust longitudinal data [1]. Second, the wide range of post-LT follow-up intervals limited the evaluation for a precise time-point post-LT or the cumulative relapse rates. Of note, the evaluation for RAULT was synchronized for both studied modalities. Third, although PEth was measured using validated LC–MS/MS protocols [27, 38], inter-laboratory variability and population-specific factors cannot be excluded. Some relapses may have gone undetected due to low alcohol intake or testing outside the PEth detection window. Although PEth was measured quarterly or semi-annually, monthly testing would likely increase sensitivity given the 2 to 4-week detection window. However, such intensive monitoring is more typical for addiction medicine follow-up and was beyond the scope of the LT center care.

The strengths of this study include its prospective design, standardized PEth testing, and rigorous side-by-side comparison of two diagnostic strategies.

## Conclusions

In this prospective study of post-LT patients with ALD, RAULT was detected in approximately one-half of patients using ClinC criteria and in one-third using PEth. Discordance between the two modalities was primarily driven by ClinC-positive/PEth-negative cases, suggesting possible overestimation by clinical criteria. In addition, PEth identified 7.7% of cases not detected by ClinC. These differences highlight the distinct detection scopes of each diagnostic approach rather than confirmatory diagnostic errors.

Quarterly PEth sampling captured more episodes of severe RAULT than semi-annual testing, although overall RAULT detection rates did not differ significantly between intervals. Time since transplantation emerged as the only independent predictor of RAULT, underscoring the need for sustained long-term monitoring.

Despite its limitations, our study provides valuable real-world data on the integration of PEth into post-LT care. Our findings support the incorporation of PEth testing into standard post-LT care pathways, offering a more comprehensive assessment of post-transplant AUD dynamics. Quarterly sampling may be recommended for patients identified as high risk for relapse, whereas semi-annual testing may represent a cost-effective strategy for clinically stable individuals.

## Supplementary Information

Below is the link to the electronic supplementary material.

Supplementary file1 (TIFF 4546 KB)

Supplementary file2 (TIFF 4546 KB)

Supplementary file3 (DOCX 15 KB)

Supplementary file4 (DOCX 15 KB)

Supplementary file5 (DOCX 15 KB)

Supplementary file6 (DOCX 15 KB)

## Abbreviations

**ALD**: Alcohol-associated liver disease
**ALT**: Alanine aminotransferase
**AST**: Aspartate aminotransferase
**AUD**: Alcohol use disorders
**BACT**: Backward alcohol capture time
**CI**: Confidence interval
**ClinC**: Clinical criteria
**CLSI**: Clinical and laboratory standards institute
**CTP**: Child–Pugh score
**DILI**: Drug-induced liver injury
**EP05-A3 / EP15-A3**: CLSI validation protocols
**GGT**: Gamma-glutamyltransferase
**HPLC–MS/MS**: High-performance liquid chromatography coupled with tandem mass spectrometry
**IQR**: Interquartile range
**LT**: Liver transplantation
**MCV**: Mean corpuscular volume
**MELD**: Model for end-stage liver disease
**OR**: Odds ratio
**PEth**: Phosphatidylethanol
**PLD**: Phospholipase D
**RAULT**: Return to alcohol use after liver transplantation
**TLFB**: Timeline follow-back
**ULN**: Upper limit of normal
