# Supplementary material for: Phosphatidylethanol versus clinical criteria to diagnose alcohol use relapse after liver transplantation: a prospective cohort study
Source: Hepatol Int. 2026 May 22;20(4):1009–19. doi: 10.1007/s12072-026-11094-4 (PMC13518525; doi:10.1007/s12072-026-11094-4)
Supplement: Supplementary file 1 [file 12072_2026_11094_MOESM3_ESM.docx]

**Supplementary Table S1. Interpretation of alcohol intake based on PEth 16:0/18:1 concentration (μg/g)**

| **PEth** | **μmol/L** | **ng/ml** | **Interpretation** |
| --- | --- | --- | --- |
|  | <0.05 | <20 | Abstinence or low alcohol intake |
|  | ≥0.05 and ≤0.30 | ≥20 and ≤200 | Moderate alcohol intake |
|  | >0.30 | ≥200 | Excessive alcohol intake |
