# Supplementary material for: Phosphatidylethanol versus clinical criteria to diagnose alcohol use relapse after liver transplantation: a prospective cohort study
Source: Hepatol Int. 2026 May 22;20(4):1009–19. doi: 10.1007/s12072-026-11094-4 (PMC13518525; doi:10.1007/s12072-026-11094-4)
Supplement: Supplementary file 2 [file 12072_2026_11094_MOESM4_ESM.docx]

**Supplementary Table S2. Frequency distribution of relapse episodes identified by PEth**

| **Number of RAULT episodes** | **Number of patients (%)** |
| --- | --- |
| 0 | 97 (67.8) |
| 1 | 8 (5.6) |
| 2 | 8 (5.6) |
| 3 | 4 (2.8) |
| 4 | 8 (5.6) |
| 5 | 9 (6.3) |
| 6 | 6 (4.2) |
| 7 | 2 (1.4) |
| 8 | 1 (0.7) |
| *RAULT: Relapse of alcohol use after liver transplantation* | |
