# Supplementary material for: Phosphatidylethanol versus clinical criteria to diagnose alcohol use relapse after liver transplantation: a prospective cohort study
Source: Hepatol Int. 2026 May 22;20(4):1009–19. doi: 10.1007/s12072-026-11094-4 (PMC13518525; doi:10.1007/s12072-026-11094-4)
Supplement: Supplementary file 3 [file 12072_2026_11094_MOESM5_ESM.docx]

**Supplementary Table S3. Permutations between ClinC and PEth diagnosed severe RAULTs in the study cohort**

|  | Severe RAULT |
| --- | --- |
| Severe RAULT, concordance | 80% |
| Sobriety, concordance | 94.3% |
| Severe RAULT, discordance  *Severe RAULT was not diagnosed by ClinC but was unveiled by PEth* | 20% |
| Sobriety, discordance  *PEth vindicated patients from severe RAULT diagnosed by ClinC* | 5.7% |
| *RAULT: Relapse of alcohol use after liver transplantation; ClinC: Clinical criteria; PEth: Phosphatidylethanol; Concordance: Diagnosis by ClinC and PEth agreed; Discordance: ClinC and PEth were at odds, PEth was considered the gold standard* | |
