# Supplementary material for: Phosphatidylethanol versus clinical criteria to diagnose alcohol use relapse after liver transplantation: a prospective cohort study
Source: Hepatol Int. 2026 May 22;20(4):1009–19. doi: 10.1007/s12072-026-11094-4 (PMC13518525; doi:10.1007/s12072-026-11094-4)
Supplement: Supplementary file 4 [file 12072_2026_11094_MOESM6_ESM.docx]

**Supplementary Table S4. Severe and moderate RAULT diagnosed by ClinC and by PEth in the study cohort**

|  | ClinC (N=69) | PEth (N=46) |
| --- | --- | --- |
| Severe RAULT (from RAULTs) | 20 (28.9%) | 23 (50%) |
| Moderate RAULT (from RAULTs) | 49 (71.7%) | 23 (50%) |
